# Gut microbiota mediates SREBP-1c-driven hepatic lipogenesis and steatosis in response to zero-fat high-sucrose diet

**DOI:** 10.1016/j.molmet.2025.102162

**Published:** 2025-05-07

**Authors:** Mattias Bergentall, Valentina Tremaroli, Chuqing Sun, Marcus Henricsson, Muhammad Tanweer Khan, Louise Mannerås Holm, Lisa Olsson, Per-Olof Bergh, Antonio Molinaro, Adil Mardinoglu, Robert Caesar, Max Nieuwdorp, Fredrik Bäckhed

**Affiliations:** 1 Wallenberg Laboratory, Department of Molecular and Clinical Medicine and Sahlgrenska Center for Cardiovascular and Metabolic Research, University of Gothenburg, Gothenburg, SE-413 45, Sweden; 2 Science for Life Laboratory, KTH - Royal Institute of Technology, Stockholm, Sweden; 3 Centre for Host-Microbiome Interactions, Faculty of Dentistry, Oral & Craniofacial Sciences, King's College London, London, SE1 9RT, UK; 4 Department of (Experimental) Vascular Medicine, Amsterdam University Medical Centers, University of Amsterdam, Amsterdam, the Netherlands; 5 Department of Clinical Physiology Region Västra Götaland, Sahlgrenska University Hospital Gothenburg Sweden, Sweden

**Keywords:** *de novo* lipogenesis, Gut microbiota, Hepatic steatosis, High-sucrose diet, Metabolomics, SREBP-1c, Zero-fat diet

## Abstract

**Objectives:**

Sucrose-rich diets promote hepatic *de novo* lipogenesis (DNL) and steatosis through interactions with the gut microbiota. However, the role of sugar-microbiota dynamics in the absence of dietary fat remains unclear. This study aimed to investigate the effects of a high-sucrose, zero-fat diet (ZFD) on hepatic steatosis and host metabolism in conventionally raised (CONVR) and germ-free (GF) mice.

**Methods:**

CONVR and GF mice were fed a ZFD, and hepatic lipid accumulation, gene expression, and metabolite levels were analyzed. DNL activity was assessed by measuring malonyl-CoA levels, expression of key DNL enzymes, and activation of the transcription factor SREBP-1c. Metabolomic analyses of portal vein plasma identified microbiota-derived metabolites linked to hepatic steatosis. To further examine the role of SREBP-1c, its hepatic expression was knocked down using antisense oligonucleotides in CONVR ZFD-fed mice.

**Results:**

The gut microbiota was essential for sucrose-induced DNL and hepatic steatosis. In CONVR ZFD-fed mice, hepatic fat accumulation increased alongside elevated expression of genes encoding DNL enzymes, higher malonyl-CoA levels, and upregulation of SREBP-1c. Regardless of microbiota status, ZFD induced fatty acid elongase and desaturase gene expression and increased hepatic monounsaturated fatty acids. Metabolomic analyses identified microbiota-derived metabolites associated with hepatic steatosis. SREBP-1c knockdown in CONVR ZFD-fed mice reduced hepatic steatosis and suppressed fatty acid synthase expression.

**Conclusions:**

Sucrose-microbiota interactions and SREBP-1c are required for DNL and hepatic steatosis in the absence of dietary fat. These findings provide new insights into the complex interplay between diet, gut microbiota, and metabolic regulation.

## Highlights

• Gut microbiota is essential for hepatic steatosis and lipogenesis in response to a high-sucrose, zero-fat diet (ZFD).
• SREBP-1c activation drives lipid synthesis and fat accumulation in the liver under ZFD conditions.
• Interaction between ZFD and gut microbiota raises portal vein levels of purine metabolites associated with hepatic steatosis.
• SREBP-1c knockdown reduces hepatic fat content and suppresses fatty acid synthase expression in ZFD-fed mice.

## 1 Introduction

Metabolic dysfunction-Associated Fatty Liver Disease (MAFLD) has increased in parallel with the global obesity epidemic and is now the most common liver disease. The initial stage of MAFLD is liver steatosis, which can lead to more severe disease stages such as metabolic associated steatohepatitis, liver fibrosis, cirrhosis and liver cancer (Fazel et al., 2016).

MAFLD is associated with an imbalanced gut microbiota [1,2], and several MAFLD-associated traits have been identified in the human microbiome [[3], [4], [5]]. In addition, hepatic steatosis can be transmitted from humans to mice through transfer of the microbiota [4], suggesting that the gut microbiota plays a causal role in the development of MAFLD.

Diets high in calories and with excessive amounts of saturated fats and sugars contribute to the development of hepatic steatosis [6]. Experiments in mice have shown that diet composition affects the role of the gut microbiota in the development of diet-induced steatosis. A Western-style diet high in saturated fat and sucrose leads to steatosis in conventionalized mice, but not in germ-free (GF) mice [7]. In contrast, high-fat diet with high content of saturated fatty acids and a low content of sucrose does not cause steatosis in either conventional or GF mice [8]. Furthermore, several studies have demonstrated that sucrose or fructose can induce steatosis in rodents in a manner dependent on gut bacteria [[9], [10], [11]]. Taken together, these observations suggest that dietary sugar interacts with the gut microbiota to induce MAFLD. Here, we use GF mice and a sucrose-rich zero-fat diet (here called ZFD) to determine how sucrose interacts with the microbiota to produce hepatic steatosis in the absence of dietary fat.

## 2 Materials and methods

### 2.1 Mice

Male C57Bl/6 mice (11–17 weeks old) were housed at 20 ± 1 °C with 45–70% humidity under a 12-hour light/dark cycle (lights on 7 a.m.–7 p.m.) in specific-pathogen-free (SPF) or GF conditions. Mice were provided ad libitum access to a sterile, irradiated high-sucrose diet (ZFD; Harlan TD.03314: 0% kcal fat, 24.2% kcal protein, 75.8% kcal sucrose) or an autoclaved chow diet (LabDiet, St. Louis, MO, USA) along with sterile water. The mice were fasted 4 h before blood samples were collected under deep isoflurane anesthesia and the mice were euthanized.

### 2.2 Liver histology

Liver biopsies were fixed in 4% paraformaldehyde (PFA) in PBS for 24 h, followed by cryoprotection in 10% and 20% sucrose solutions in PBS for 12 h each. Tissue sections were prepared at HistoCenter (Gothenburg, Sweden). Neutral lipids were stained with Oil Red O using a Leica Autostainer or manual methods and visualized using a Zeiss Axio Imager M1 microscope. Digital image acquisition was performed using Axiovision software (Zeiss, Germany).

### 2.3 Liver triglycerides

Hepatic triglycerides were extracted using the semi-automated BUME method for lipid analysis [12]. The extracts were diluted in chloroform (1:2) containing 5 mM ammonium acetate and analyzed via direct infusion mass spectrometry [13] using a TriVersa NanoMate (Advion BioSciences, Ithaca, NY) coupled to a QTRAP 5500 mass spectrometer (ABSciex, Canada).

### 2.4 Gene expression analyses

RNA was isolated from snap-frozen liver tissue using the RNeasy kit (Qiagen, Germany), including on-column DNase treatment. RNA integrity was assessed using a Bioanalyzer (Agilent Technologies), with RIN values ranging from 9.3 to 10.0. RNA sequencing was conducted at Science for Life Laboratory (Stockholm, Sweden) using Illumina TruSeq RNA libraries. Sequencing was performed on an Illumina HiSeq2500 platform with single-end 50 bp reads, generating over 680 million reads. Reads were trimmed (Phred score ≥15; minimum length 40 bp) using the Fastx-toolkit and mapped to the *Mus musculus* GRCm38 genome with TopHat v2.0.4 using the Bowtie2 aligner. Gene counts were obtained using HTSeq-count.

For qRT-PCR, cDNA synthesis was performed with the High-Capacity cDNA Reverse Transcription Kit (Applied Biosystems) using 0.5 μg RNA. Gene expression was quantified with SYBR Green-based PCR (Thermo Scientific, Waltham, MA) and normalized to Rpl32. Primer sequences are provided in Supplementary Table 5.

### 2.5 Malonyl-CoA quantification

Hepatic malonyl-CoA levels were quantified using a mouse malonyl-CoA ELISA kit (MyBioSource, San Diego, CA) according to the manufacturer's protocol.

### 2.6 SREBP-1c knockdown

*Srebf1*-specific antisense oligonucleotides (ASOs; sequence: CCAGATCTGCCACTAGAGGT) were prepared at a concentration of 2.5 mg/mL in sterile PBS, filtered, and stored at −20 °C. Mice received intraperitoneal injections of 25 mg/kg ASO twice weekly for three weeks, with injection volumes not exceeding 250 μL.

### 2.7 Metabolomics

Portal vein blood samples were collected into EDTA tubes, centrifuged at 10,000 rpm for 5 min, and the plasma supernatant was stored at −80 °C. Chromatography and mass spectrometry analyses were performed by Metabolome Inc., and metabolic pathway integration was visualized using the Cytoscape MetaboLync plugin.

### 2.8 16S rRNA profiling of the cecal microbiota

Genomic DNA was extracted from cecum samples of mice fed ZFD (n = 9) or chow diet (n = 8), and approximately 50 ng of template DNA were amplified in duplicate reactions as previously described [14] using dual-indexed primers 515F and 806R [15] targeting the V4 region of the 16S rRNA gene. Amplicons were sequenced in an Illumina Miseq instrument using the V2 kit (2 × 250 bp paired-end reads). Raw paired-end reads were processed using QIIME 2 (version 2024.10) [16] Quality profiles of the raw sequences were assessed with FastQC (v0.12.1) [17] and aggregated using MultiQC [18] to determine optimal trimming parameters. Denoising was performed using DADA2 [19] with forward and reverse reads truncated at 200 bp and 180 bp, respectively. This step generated a feature table, representative sequences, and denoising statistics. Taxonomic classification of amplicon sequence variants (ASVs) was conducted using a pre-trained Naive Bayes classifier based on the SILVA 138 reference database [20,21]. The feature table was subsequently collapsed to genus level to calculate taxon-wise relative abundances, resulting in 104 genera included in the analyses. Graphical representations and statistical analyses of gut microbiota profiles were performed using R v.4.4.3 [22] with packages vegan v.2.6–10 [23] and ggplot2 v.3.5.1 [24]. Genus-level abundance data were rarefied to the minimum sequencing depth across samples using the vegan package; α-diversity was computed using the Shannon diversity index and β-diversity was computed using the Bray–Curtis distance.

### 2.9 Statistical analyses

Statistical analyses of RNA-seq and metabolomics were performed using R Version 4.4.1 (R Foundation for Statistical Computing, Vienna, Austria), R package rstatix 0.72 was used for two-way ANOVA analyses followed by Tukey's post hoc test. False discovery rate correction was performed to calculate adjusted p-values. vegan 2.6–8 for ADONIS, ropls 1.36.0 was used for PLS analyses. Analysis of enrichment of regulated genes within functional categories gene ontology categories (GO) [25] was performed using the software David [26]. The results of the enrichment calculation were filtered for GO categories that were significantly enriched (FDR <0.01) Other statistical analyses were performed using GraphPad Prism (v10.2.3). Unpaired two-tailed Student's *t*-tests were used for pairwise comparisons. Two-way ANOVA followed by Tukey's post hoc test was used for comparisons involving multiple factors. For gut microbiota analyses, differences in α-diversity were assessed using the Wilcoxon rank sum test. Differential abundance of ASVs collapsed at genus level was assessed using MaAsLin2 [27] and the Benjamini-Hochberg procedure was used to adjust for false discovery rate [28]; significance was defined for features with an adjusted p-value <0.01. Differences in β-diversity were assessed using the adonis function [29,30] in vegan v.2.6–10 [23].

### 2.10 Data availability

The sequencing data generated in this study has been deposited in the European Nucleotide Archive (ENA) under the project number **PRJEB85296**.

## 3 Results

### 3.1 ZFD diet induces hepatic steatosis and DNL in the presence of a gut microbiota

**Figure 1:**
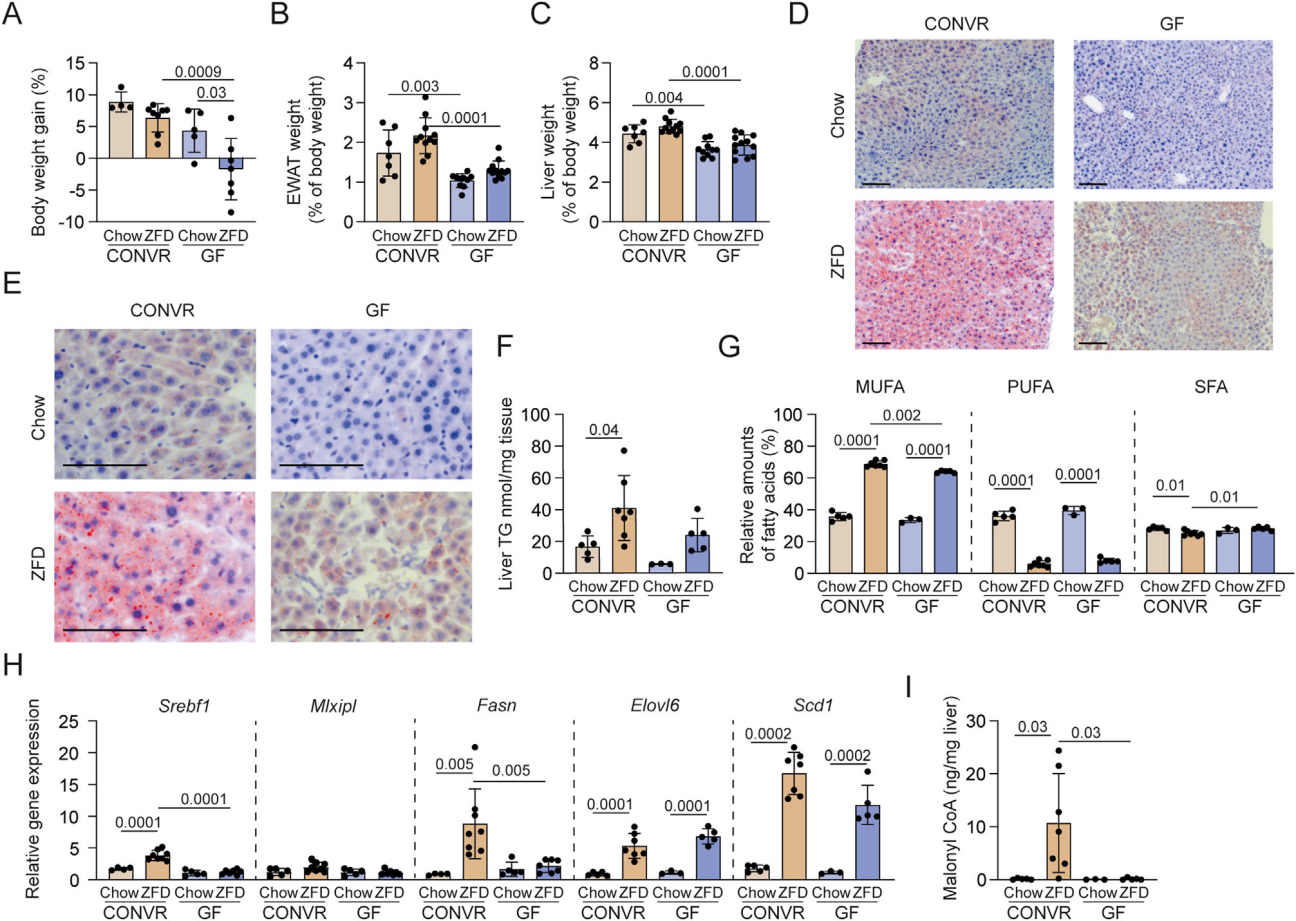
**The gut microbiota contributes to ZFD-induced steatosis.** Mice were fed ZFD or chow diet for 3 weeks. (A) Body weight gain after diet intervention (n = 4–8/group). (B) Relative weight of EWAT and (C) liver (n = 7–12/group). (D–E) Representative micrographs of Oil Red O staining for neutral lipids. Scale bar = 200 μm. (F) Liver triglyceride-derived fatty acids and (G) relative amounts of SFA, MUFA, and PUFA (n = 3–7/group). (H) Hepatic gene expression determined by qRT-PCR (n = 3–8/group). (I) Liver concentrations of malonyl-CoA (n = 3–7/group). Significant *p* values for diet and colonization status were determined by two-way ANOVA with Tukey's multiple comparisons. Data are presented as mean ± SD. Abbreviations: CONVR – conventionally raised; GF – germ-free; ZFD – zero-fat diet; SFA – saturated fatty acids; MUFA – monounsaturated fatty acids; PUFA – polyunsaturated fatty acids; *Srebf1* – sterol regulatory element binding transcription factor 1; *Fasn* – fatty acid synthase; *Elovl6* – fatty acid elongase 6; *Scd1* – stearoyl-Coenzyme A desaturase 1. (For interpretation of the references to color in this figure legend, the reader is referred to the Web version of this article.)

To assess how the interaction between a high-sucrose, fat-free diet (ZFD; 75.8% kcal sucrose) and gut microbiota influences hepatic steatosis and metabolism, we fed conventionally raised (CONVR) and GF mice either ZFD or standard chow for 3 weeks. CONVR and GF mice on chow, as well as CONVR mice on ZFD, gained 5–10% body weight, whereas GF mice on ZFD did not gain weight (Figure 1A). Relative weights of epididymal white adipose tissue (EWAT) and liver were significantly lower in GF mice compared to CONVR mice on both diets (Figure 1B and C).

Neutral lipid staining and triglyceride quantification using mass spectrometry revealed increased hepatic fat accumulation in CONVR mice fed ZFD (Figure 1D–F, Supplementary Table 1). Hepatic triglyceride levels of saturated fatty acids (SFA) correlated with steatosis severity (Supplementary Table 1). Meanwhile, the triglycerides in ZFD-fed mice exhibited increased proportions of monounsaturated fatty acids (MUFA) and decreased polyunsaturated fatty acids (PUFA) (Figure 1G). This shift in fatty acid composition is expected, as ZFD lacks PUFA, which cannot be synthesized *de novo* by mammals. The gut microbiota increased MUFA levels and reduced the proportion of SFA in hepatic triglycerides from mice fed ZFD, but not in those fed a chow diet. SFA was significantly affected by the interaction between diet and microbiota (*p* = 0.006).

Building on the established role of hepatic *de novo* lipogenesis (DNL) in MAFLD [31], we analyzed the expression of key genes involved in lipogenesis. qRT-PCR analysis revealed that ZFD-fed CONVR mice exhibited significantly higher expression of *Srebf1* (encoding SREBP1c), a major transcriptional regulator of DNL [32] (Figure 1H). *Srebf1* was significantly affected by the interaction between diet and microbiota (*p* = 0.0006). In contrast, the expression of *Mlxipl* (encoding ChREBP, another key transcriptional regulator of DNL [33]) remained unchanged. The expression of *Fasn* (encoding fatty acid synthase) was significantly influenced by diet–microbiota interaction (*p* = 0.006) and was elevated in response to ZFD compared to chow, with further increases observed in the presence of a gut microbiota in ZFD-fed mice. Similarly, the expression of *Elovl6* and *Scd1* (encoding fatty acid elongase 6 and stearoyl-CoA desaturase-1, respectively) was higher in ZFD-fed mice, although presence of a gut microbiota had no impact on these genes. Notably, hepatic malonyl-CoA levels, a critical substrate for fatty acid synthesis, were substantially elevated in ZFD-fed CONVR mice (Figure 1I).

In summary, our results show that ZFD promotes hepatic steatosis and DNL in the presence of a gut microbiota while also stimulating fatty acid elongation and desaturation independent of microbial colonization.

### 3.2 Interaction between ZFD and the gut microbiota regulates hepatic lipid metabolism

**Figure 2:**
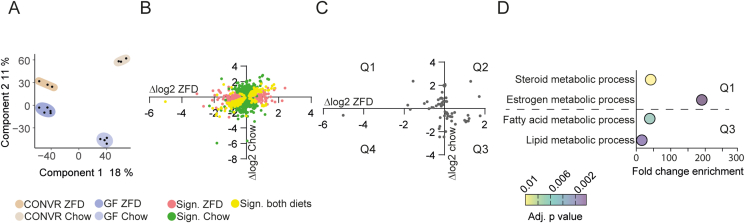
**Interaction between ZFD and gut microbiota regulates hepatic lipid metabolism.** RNA-seq analysis was performed on liver tissue from CONVR (n = 3/diet) and GF (n = 5/diet) mice fed ZFD or chow diet for 3 weeks. (A) Partial least squares (PLS) analysis of liver RNA-seq data, showing sample separation by microbiota status and diet. Each point represents an individual sample. Axes indicate variance explained by the first two PLS components. (B) Microbiota-induced gene regulation in mice fed ZFD (**x**-axis) or chow (**y**-axis). (C) Genes regulated by the interaction between diet and gut microbiota. Microbiota-induced regulation in mice fed ZFD (**x**-axis) or chow (**y**-axis). (D) Gene ontology categories enriched in subsets of genes located in quadrants Q1 and Q3 in panel (C). Statistical analysis was performed using a two-way ANOVA followed by Tukey's HSD post-hoc test. p-values were adjusted using false discovery rate (FDR) correction, and a corrected p-value <0.05 was considered statistically significant and are displayed in B and C. Abbreviations: CONVR – conventionally raised; GF – germ-free; ZFD – zero-fat diet.

To further explore how ZFD and the gut microbiota interact to regulate hepatic physiology, we performed transcriptome analysis on liver samples from CONVR and GF mice using RNA-seq. Partial Least Squares Discriminant Analysis (PLS-DA) revealed distinct sample clustering, with diet accounting for 18% of variance along the first dimension and microbiota contributing 12% along the second dimension (Figure 2A).

To assess microbiota-mediated regulation of hepatic gene expression, we compared significantly regulated genes between CONVR and GF mice. 1,213 genes were regulated by the microbiota in chow-fed mice, 544 in ZFD-fed mice, and 234 across both diets, indicating that microbiota-driven gene regulation is largely diet-dependent (Figure 2B).

Gene ontology enrichment analysis revealed that genes associated with xenobiotic and steroid metabolism were regulated by the microbiota in chow-fed mice (Supplementary Table 2), whereas lipid metabolic processes were regulated across both diets. In ZFD-fed mice, the microbiota upregulated genes involved in glucose metabolism and downregulated genes associated with immune responses, a trend observed across both diets.

Using two-way ANOVA, we identified genes whose expression was modulated by the interaction between diet and gut microbiota (Figure 2C). Genes in Q1 (upregulated by the microbiota in chow-fed mice and downregulated in ZFD-fed mice) were enriched for pathways related to steroid and estrogen metabolism. In contrast, genes in Q3 (upregulated by the microbiota in ZFD-fed mice and downregulated in chow-fed mice) were enriched for pathways associated with fatty acid and lipid metabolism (Figure 2D).

RNA-seq analysis was in agreement with the expression data from Figure 1H and revealed regulation of glucose and lipid transporters. In addition, ZFD increased the expression of the long chain free fatty acid transporter *Cd36* in both GF and CONVR mice, consistent with prior studies on high-sugar diets [34,35] (Supplementary Figure 1A). *Cd36* was also elevated in GF compared with CONVR mice fed chow. *Slc2a2*, encoding the sugar-transporter GLUT2, was significantly increased in CONVR, but not in GF, mice fed ZFD, correlating with the observed rise in *de novo* lipogenesis (Supplementary Figure 1B).

These findings demonstrate that the gut microbiota interacts with diet to modulate hepatic gene expression and influences lipid and glucose metabolism as well as immune responses in a diet-dependent fashion.

### 3.3 Interaction between ZFD and the gut microbiota regulates portal vein plasma metabolome

The gut microbiota communicates with the host via bioactive metabolites, influencing key metabolic pathways [36]. To investigate how diet and microbiota affect the metabolome, we performed untargeted metabolomics on portal vein plasma.

**Figure 3:**
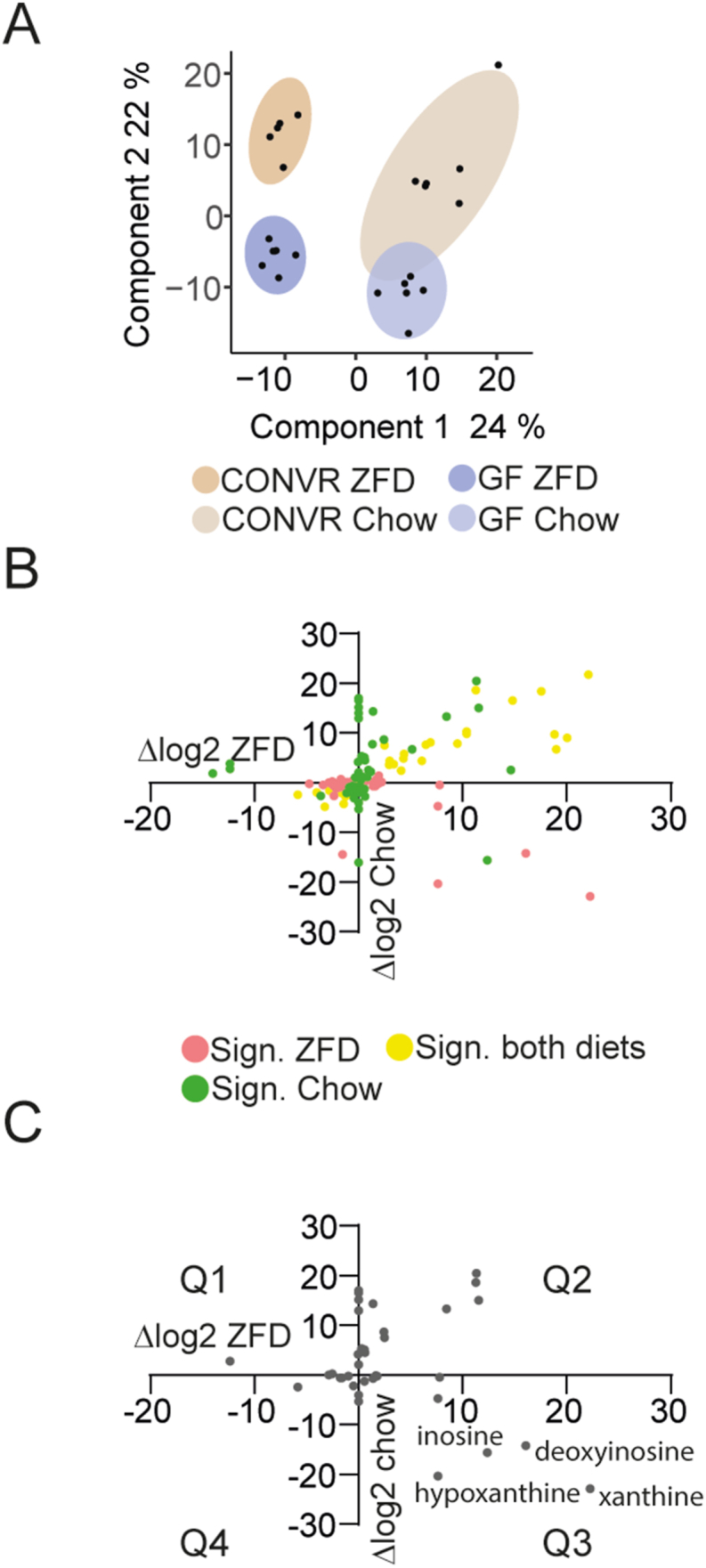
**Interaction between ZFD and gut microbiota regulates the portal vein metabolome.** Analysis was performed on plasma from CONVR and GF mice fed ZFD or chow diet for 3 weeks (n = 5–6/group). (A) Partial least squares (PLS) analysis based on 536 compounds, showing sample separation by microbiota status and diet. Each point represents an individual sample. Axes indicate variance explained by the first two PLS components. (B) Microbiota-induced metabolite regulation in mice fed ZFD (**x**-axis) or chow (**y**-axis). (C) Metabolites regulated by the interaction between diet and gut microbiota. Microbiota-induced regulation in mice fed ZFD (**x**-axis) or chow (**y**-axis). Statistical analysis was performed using a two-way ANOVA followed by Tukey's HSD post-hoc test. p-values were adjusted using false discovery rate (FDR) correction, and a corrected p-value <0.05 was considered statistically significant and are displayed in B and C. Abbreviations: CONVR – conventionally raised; GF – germ-free; ZFD – zero-fat diet.

PLS-DA analysis of metabolite profiles showed a clear separation of samples, with diet explaining 24% of variance along the first dimension and the microbiota contributing 22% along the second dimension (Figure 3A). We identified 536 metabolites, including 16 metabolites increased and 42 decreased by the microbiota exclusively in ZFD-fed mice, 30 increased and 33 decreased exclusively in chow-fed mice, and 20 increased and 23 decreased across both diets (Figure 3B, Supplementary Table 3). Two-way ANOVA identified metabolites modulated by the interaction between diet and microbiota (Figure 3C). Notably, metabolites in Q3 (upregulated by the microbiota in ZFD-fed mice and downregulated in chow-fed mice), including inosine, deoxyinosine, hypoxanthine, and xanthine, which are intermediates in purine metabolism.

These findings highlight the interplay between diet, the gut microbiota, and the portal plasma metabolome, with a particular focus on purine metabolism regulation.

### 3.4 ZFD and chow diets induce distinct gut microbiota compositions

To assess the impact of the diet on gut microbiota, we performed 16S rRNA gene sequencing and compared overall composition, species diversity and abundance of taxa in in cecal samples from ZFD and Chow fed mice. Principal Coordinate Analysis (PCoA) on Bray–Curtis distance showed a clear separation (Adonis R^2^ = 0.49, p = 0.001) between the ZFD and Chow groups indicating distinct compositions (Supplementary Fig. 2A). α-diversity measured by the Shannon index did not differ significantly between the groups (Wilcoxon Rank Sum Test, p = 0.54; Supplementary Fig. 2B). Several taxa were differentially abundant, with distinct enrichments in each group and correlations with liver and metabolic parameters (Supplementary Fig. 2C).

### 3.5 ZFD induces hepatic lipogenesis and steatosis in conventionally raised mice through SREBP-1c activation

**Figure 4:**
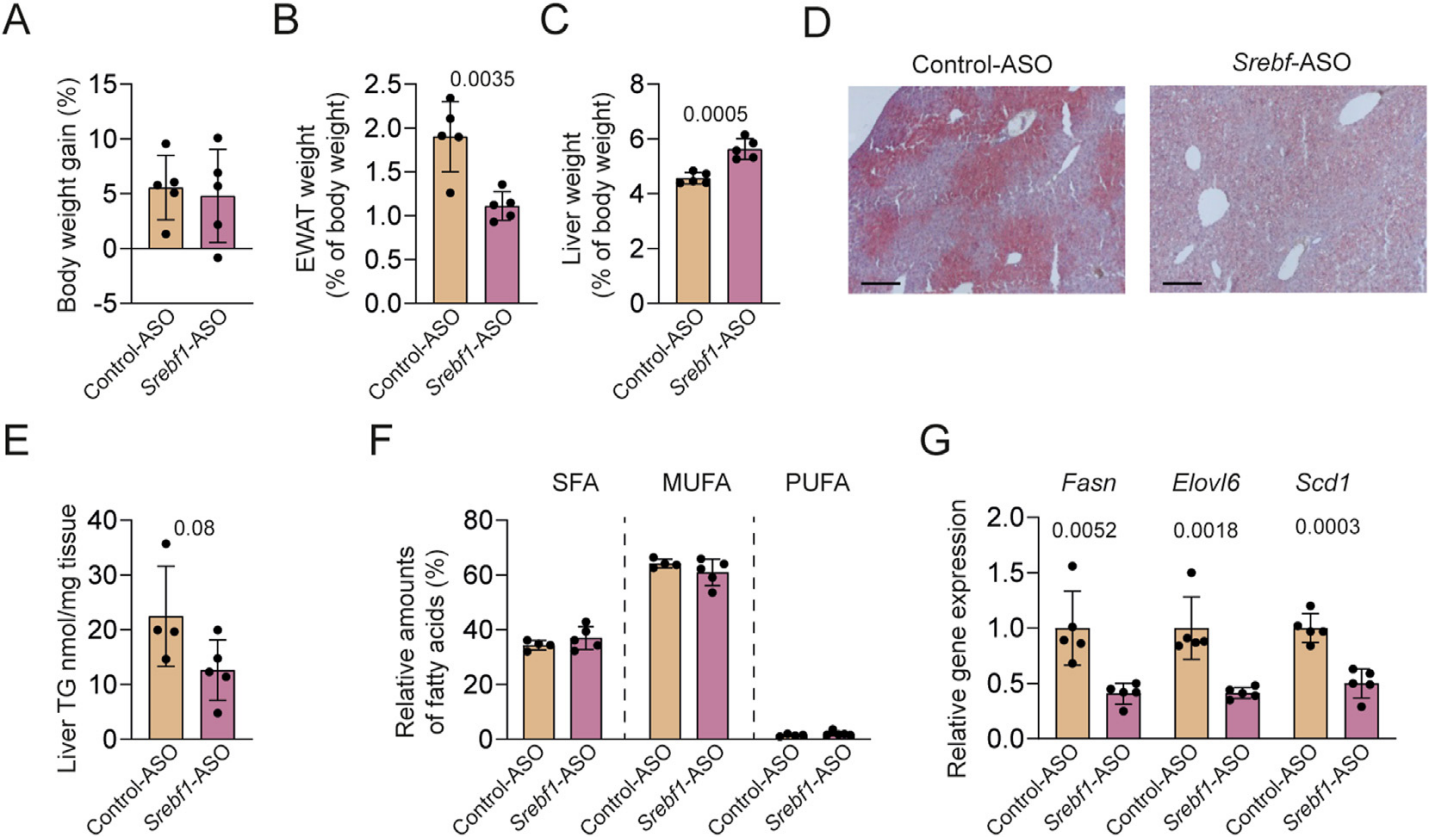
***Srebf1* knockdown partially prevents ZFD-induced hepatic steatosis in CONVR mice.** Mice were treated with *Srebf1*-specific ASO for 3 weeks and fed ZFD (n = 4–5/group). (A) Body weight gain, (B) relative EWAT weight, and (C) liver weight. (D) Representative micrographs of Oil Red O staining for neutral lipids. Scale bar = 200 μm. (E) Liver triglyceride-derived fatty acids and (F) relative amounts of SFA, MUFA, and PUFA. (G) Hepatic gene expression determined by qRT-PCR. Significant *p* values were determined by Student's *t*-test. Data are presented as mean ± SD. Abbreviations: *Srebf1* – sterol regulatory element binding transcription factor 1; ASO – antisense oligonucleotide; SFA – saturated fatty acids; MUFA – monounsaturated fatty acids; PUFA – polyunsaturated fatty acids. (For interpretation of the references to color in this figure legend, the reader is referred to the Web version of this article.)

To confirm the role of SREBP-1c in ZFD-induced lipogenesis and steatosis, we treated ZFD-fed CONVR mice with *Srebf1*-specific antisense oligonucleotides (ASO) for three weeks, causing a reduction of hepatic *Srebf1* expression by 70% (Supplementary Figure 3A). ASO treatment was well-tolerated, with no effect on body weight gain (Figure 4A) or *Tnfα* expression (Supplementary Figure 3B). However, the treatment reduced relative EWAT weight (Figure 4B) and increased relative liver weight (Figure 4C). Neutral lipid staining revealed reduced hepatic fat in ASO-treated mice (Figure 4D), accompanied by a trend towards lower triglyceride-derived fatty acid content (p = 0.08; Figure 4E). No major differences in fatty acid composition were observed (Figure 4F and Supplementary Table 4). Expression of *Fasn*, *Elovl6*, and *Scd1* was significantly reduced in ASO-treated mice, supporting decreased lipogenesis (Figure 4G).

Together, these data indicate that ZFD-induced hepatic steatosis in CONVR mice is mediated by SREBP-1c activation, which drives lipogenesis and alters lipid metabolism.

## 4 Discussion

Here we demonstrate that gut microbiota promotes DNL and steatosis when fed high levels of sucrose in the absence of dietary fat through, at least in part, a SREBP-1c dependent pathway. We also show that ZFD promotes elongation and desaturation of fatty acids independent of colonization status, resulting in increased hepatic levels of MUFA. Our findings reveal that the interaction between diet and microbiota influences hepatic lipid metabolism as well as portal vein metabolite levels, including metabolites involved in purine metabolism. These results underscore the complex interplay between dietary composition, microbial activity, and host metabolic pathways, highlighting the gut microbiota's role as a key modulator of metabolic outcomes.

Consistent with our findings, previous studies have established that high-sugar diets, including those rich in fructose or sucrose, contribute to hepatic steatosis in both humans and mice [[37], [38], [39]]. Specifically, Chakravarthy et al. [40] showed that a fat-free, high-sugar diet induces steatosis in mice without changes in body weight, liver weight, or adiposity. These observations align with the notion that sugar-induced steatosis is driven primarily by metabolic dysregulation rather than excessive calorie intake. Our findings further build on this observation by demonstrating the additional influence of the gut microbiota, suggesting that microbial factors may amplify or modify the lipogenic response to dietary sugar. Todoric et al. [41] further demonstrated that fructose disrupts intestinal barrier integrity, leading to endotoxemia and hepatic *Tnfα* expression, which activates SREBP-1c, promoting lipogenesis. While our findings also implicate SREBP-1c in lipogenesis, we did not observe increased hepatic expression of TNFα or other proinflammatory genes, suggesting the involvement of alternative pathways. Furthermore, Zhao et al. [42] highlighted the role of gut microbiota in converting fructose into acetate, which is transported to the liver and used as a substrate for lipogenesis. However, our study found that the activation of lipogenic genes in CONVR mice fed ZFD was not driven by gut microbiota-derived acetate. This discrepancy points to other microbiota-mediated mechanisms influencing lipogenesis.

We also find that ZFD induces steatosis in GF mice, possibly due to low PUFA levels, which can increase SREBP-1c activity [43] and decrease PPARα activity [44], leading to increased expression of lipogenic genes like *Elovl6* and *Scd1* and a shift toward lipid synthesis.

We identified metabolites uniquely upregulated in ZFD-fed CONVR mice, including several purine pathway metabolites. Elevated purine metabolites are linked to increased xanthine oxidoreductase (XOR) activity, which catalyzes their conversion to uric acid [45]. XOR activity has been associated with hepatic oxidative stress and MAFLD development [46]. and recent studies demonstrated that gut bacterial metabolism influences host purine homeostasis [47]. It has also been shown that excessive fructose intake causes lipid accumulation and triglyceride synthesis via the purine degradation pathway [48]. However, it remains to be demonstrated if the purine metabolites identified in this study drive or result from increased steatosis. It also remains to be investigated if the altered gut microbiota following ZFD contributes to the regulation of purine metabolism.

The transcriptional regulation of lipogenesis involves several factors, with SREBP-1c being a key player [49]. Our findings show that *Srebf1* expression is increased in CONVR mice fed ZFD, and its knockdown reduces both *Fasn* expression and liver fat content. Notably, this intervention did not affect body weight, aligning with prior observations that reducing steatosis can occur independently of changes in body or adipose tissue weight [50].

We observed a significant increase in the DNL precursor malonyl-CoA in CONVR mice fed ZFD. Malonyl-CoA acts as a potent steric inhibitor of carnitine palmitoyltransferase 1 (CPT1), limiting lipid transport into mitochondria for β-oxidation (McGarry et al., 1978). This inhibition may contribute to elevated hepatic lipid content by suppressing fatty acid oxidation.

Taken together this study underscores the critical role of the gut microbiota in mediating the metabolic effects of a high-sucrose, fat-free diet, including its impact on lipogenesis and hepatic lipid metabolism. By identifying SREBP-1c as a central regulator of diet- and microbiota-induced lipogenesis, our findings provide valuable insights into the mechanisms underlying diet-induced steatosis.

## CRediT authorship contribution statement

**Mattias Bergentall:** Writing – original draft, Investigation, Formal analysis. **Valentina Tremaroli:** Writing – review & editing, Investigation. **Chuqing Sun:** Formal analysis, Writing – review & editing. **Marcus Henricsson:** Writing – review & editing, Investigation, Formal analysis. **Muhammad Tanweer Khan:** Writing – review & editing, Investigation. **Louise Mannerås Holm:** Writing – review & editing, Investigation, Conceptualization. **Lisa Olsson:** Writing – review & editing, Formal analysis. **Per-Olof Bergh:** Writing – review & editing, Investigation, Formal analysis. **Antonio Molinaro:** Writing – review & editing, Investigation. **Adil Mardinoglu:** Writing – review & editing, Formal analysis. **Robert Caesar:** Writing – review & editing, Formal analysis, Data curation. **Max Nieuwdorp:** Writing – review & editing, Resources, Funding acquisition. **Fredrik Bäckhed:** Writing – original draft, Supervision, Resources, Project administration, Funding acquisition, Conceptualization.

## Institutional review board statement

All experimental protocols were approved by the Gothenburg Ethics Committee for Animal Care and Use.

## Funding

This study was supported by the 10.13039/501100009708Novo Nordisk Foundation, the 10.13039/501100004359Swedish Research Council, Torsten Söderberg's Foundation, Swedish Diabetes Foundation, Swedish Heart Lung Foundation, Göran Gustafsson's Foundation, IngaBritt och Arne Lundberg's Foundation, 10.13039/501100004063Knut and Alice Wallenberg Foundation, the Swedish Foundation for Strategic Research, the regional agreement on medical training and clinical research (ALF) between Region Västra Götaland and 10.13039/501100005754Sahlgrenska University Hospital. M.N. is supported by a NWO-ZonMw Vici grant 2020 (09150182010020) and an ERC-Advanced grant 2023 (101141346-FATGAP). F.B. is Wallenberg Fellow and recipient of an ERC-Advanced grant 2022 (101096705-IMPACT). The computations were enabled by resources in project NAISS 2024/5-548 provided by the National Academic Infrastructure for Supercomputing in Sweden (NAISS), partially funded by the 10.13039/501100004359Swedish Research Council through grant agreement no. 2022-06725.

## Declaration of competing interest

V.T. is co-founders and shareholders of Roxbiosens Inc. M.N is co-founder and member of the Scientific Advisory Board of Caelus Pharmaceuticals and Advanced Microbial Interventions, both spin-outs of AUMC-UvA, Amsterdam, the Netherlands. F.B. is co-founder and shareholder of Roxbiosens Inc and Implexion Pharma AB, receives research funding from Biogaia AB and Novo Nordisk A/S, and is a member of the scientific advisory board of Bactolife A/S. None of these are directly relevant to the current paper.

## Data Availability

Data will be made available on request.
