# Supplementary material for: Gut microbiota mediates SREBP-1c-driven hepatic lipogenesis and steatosis in response to zero-fat high-sucrose diet
Source: Mol Metab. 2025 May 7;97:102162. doi: 10.1016/j.molmet.2025.102162 (PMC12145984; doi:10.1016/j.molmet.2025.102162)
Supplement: Multimedia component 1 [file mmc1.pdf]

Supplementary Table 1. Concentration of hepatic triglyceride-derived fatty acids (nmol/mg) in CONVR and GF mice fed chow diet or ZFD diet. Related to Figure 1.

	**CONVR**		**GF**		**Two-way ANOVA**
**Fatty acid**	**Chow**	**ZFD**	**Chow**	**ZFD**	**p values for Diet/Microbiome/Interaction**
**14:0**	0,09 ± 0,04	0,46 ± 0,28$^{0.003}$	0,03 ± 0,01	0,25 ± 0,12	0.003/ns/ns
**14:1**	0,02 ± 0,01	0,09 ± 0,05$^{0.003}$	0,01 ± 0	0,05 ± 0,03	0.003/ns/ns
**16:0**	4,24 ± 1,82	8,87 ± 5,14$^{0.004}$	1,3 ± 0,11	5,88 ± 2,61	0.01/ns/ns
**16:1**	0,78 ± 0,44	4,94 ± 3,38$^{0.006}$	0,17 ± 0,04	3,05 ± 1,8	0.006/ns/ns
**16:2**	0,05 ± 0,02	0,1 ± 0,06	0,02 ± 0	0,06 ± 0,04	0.03/ns/ns
**18:0**	0,36 ± 0,14	1,14 ± 0,47$^{0.0006}$	0,17 ± 0,01	0,54 ± 0,2$^{0.005}$	0.001/0.02/ns
**18:1**	5,15 ± 2,35	21,94 ± 9,9$^{0.0006}$	1,65 ± 0,05	11,74 ± 4,91$^{0.02}$	0.0006/0.04/ns
**18:2**	4,2 ± 1,38	1,27 ± 0,63$^{0.0001}$	1,33 ± 0,09$^{0.0002}$	0,9 ± 0,39	0.0004/0.0005/0.005
**18:3**	0,41 ± 0,12	0,15 ± 0,03$^{0.0001}$	0,14 ± 0,02$^{0.0001}$	0,12 ± 0,05	0.0004/0.0002/0.001
**20:0**	0,02 ± 0,01	0,09 ± 0,05$^{0.003}$	0,01 ± 0	0,02 ± 0,01$^{0.004}$	0.01/0.02/ns
**20:1**	0,12 ± 0,06	1,09 ± 0,54$^{0.002}$	0,05 ± 0,01	0,39 ± 0,14$^{0.003}$	0.0008/0.03/ns
**20:2**	0,05 ± 0,03	0,24 ± 0,11$^{0.0005}$	0,03 ± 0	0,15 ± 0,05$^{0.04}$	0.0004/ns/ns
**20:3**	0,08 ± 0,04	0,28 ± 0,11$^{0.001}$	0,05 ± 0,01	0,32 ± 0,11$^{0.0006}$	0.0001/ns/ns
**20:4**	0,21 ± 0,06	0,07 ± 0,03$^{0.0001}$	0,15 ± 0,01	0,11 ± 0,03	0.0001/0.02/ns
**20:5**	0,14 ± 0,05	0,02 ± 0$^{0.0001}$	0,05 ± 0,01$^{0.0006}$	0,01 ± 0	0.0001/0.003/0.006
**22:6**	0,72 ± 0,27	0,14 ± 0,07$^{0.0001}$	0,44 ± 0,08$^{0.02}$	0,15 ± 0,07$^{0.02}$	0.0001/ns/ns
**Total**	16,65 ± 6,75	40,89 ± 20,4$^{0.009}$	5,59 ± 0,17	23,76 ± 10,48	0.005/0.05/ns

n = 5 (CONVR/Chow); 7 (CONVR/ZFD); 3 (GF/Chow); 5 (GF/ZFD). Significance for multiple comparison between CONVR/Chow vs. CONVR/ZFD - blue; CONVR/Chow vs. GF/Chow – purple; CONVR/ZFD vs. GF/ZFD – red; GF/Chow vs. GF/ZFD – green as determined by Two-way ANOVA. Data are presented as mean ± SD.
