# Supplementary material for: Gut microbiota mediates SREBP-1c-driven hepatic lipogenesis and steatosis in response to zero-fat high-sucrose diet
Source: Mol Metab. 2025 May 7;97:102162. doi: 10.1016/j.molmet.2025.102162 (PMC12145984; doi:10.1016/j.molmet.2025.102162)
Supplement: Multimedia component 2 [file mmc2.pdf]

Supplementary Table 2. Gene ontology categories enriched in genes regulated by the gut microbiota in mice fed chow or ZFD for 3 weeks. Related to Figure 2.

GO term	Description	Enrichment	FDR
Categories enriched in genes upregulated by gut microbiota in chow-fed mice			
6805	xenobiotic metabolic process	10.9	6.7x10^{-11}
8210	estrogen metabolic process	22.4	2.9x10^{-9}
6629	lipid metabolic process	3.3	6.7x10^{-8}
8202	steroid metabolic process	7.3	1.1x10^{-6}
52695	cellular glucuronidation	24.4	9.8x10^{-6}
2933	lipid hydroxylation	43.4	3.5x10^{-5}
42178	xenobiotic catabolic process	18.5	5.9x10^{-5}
6915	apoptotic process	2.9	5.9x10^{-5}
36503	ERAD pathway	7.7	1.3x10^{-4}
6749	glutathione metabolic process	10.2	1.4x10^{-4}
9822	alkaloid catabolic process	48.3	3.1x10^{-4}
43066	negative regulation of apoptotic process	2.7	3.5x10^{-4}
46166	glyceraldehydeE-3E-phosphate biosynthetic process	26.7	3.5x10^{-4}
42573	retinoic acid metabolic process	15.0	0.001
1666	response to hypoxia	4.3	0.0011
6914	autophagy	4.2	0.0014
1525	angiogenesis	3.5	0.0025
51085	chaperone cofactorE-dependent protein refolding	11.9	0.0032
6096	glycolytic process	9.3	0.0032
70989	oxidative demethylation	26.3	0.0035
6094	gluconeogenesis	8.9	0.0038
6879	intracellular iron ion homeostasis	8.3	0.0059
7040	lysosome organization	8.1	0.0063
8203	cholesterol metabolic process	5.1	0.0072
Categories enriched in genes downregulated by gut microbiota in chow-fed mice			
6629	lipid metabolic process	3.8	1x10^{-14}
6694	steroid biosynthetic process	13.8	3.1x10^{-14}
8202	steroid metabolic process	8.0	1.3x10^{-10}
16126	sterol biosynthetic process	19.6	5.6x10^{-10}
19373	epoxygenase P450 pathway	16.4	5.2x10^{-9}
8203	cholesterol metabolic process	7.1	9.1x10^{-8}
6695	cholesterol biosynthetic process	13.4	3.6x10^{-7}
8152	metabolic process	5.0	6.1x10^{-7}
6805	xenobiotic metabolic process	7.1	2.5x10^{-6}
45087	innate immune response	3.1	2.7x10^{-6}
2376	immune system process	2.9	4x10^{-5}
6869	lipid transport	4.5	3.3x10^{-4}
15990	electron transport coupled proton transport	39.0	6.2x10^{-4}
6696	ergosterol biosynthetic process	23.4	6.2x10^{-4}
6397	mRNA processing	3.0	0.0015
8380	RNA splicing	3.1	0.0046
Categories enriched in genes upregulated by gut microbiota in ZFD-fed mice			
6629	lipid metabolic process	4.1	4.9x10^{-7}
6096	glycolytic process	20.4	1x10^{-6}
46166	glyceraldehydeE-3E-phosphate biosynthetic process	47.1	5.8x10^{-5}
5975	carbohydrate metabolic process	6.9	1.8x10^{-4}
6094	gluconeogenesis	15.7	2.4x10^{-4}
6099	tricarboxylic acid cycle	19.8	3.5x10^{-4}
6098	pentoseE-phosphate shunt	39.2	0.0014
6739	NADP metabolic process	36.4	0.0017
61621	canonical glycolysis	30.0	0.0035
8152	metabolic process	5.2	0.0089
14070	response to organic cyclic compound	8.2	0.0089
Categories enriched in genes downregulated by gut microbiota in ZFD-fed mice			
45087	innate immune response	5.4	1.2x10^{-8}
2376	immune system process	4.5	8.0x10^{-6}
19373	epoxygenase P450 pathway	23.7	2.0x10^{-5}
6629	lipid metabolic process	3.2	0.0016
6805	xenobiotic metabolic process	9.3	0.0016
6954	inflammatory response	4.3	0.00166
6958	complement activation. classical pathway	13.0	0.0037
10466	negative regulation of peptidase activity	7.8	0.0044
Categories enriched in genes modulated by diet-microbiota interaction			
8210	estrogen metabolic process	206.5	1.7x10^{-14}
8202	steroid metabolic process	47.4	2.2x10^{-9}
9822	alkaloid catabolic process	592.8	5.8x10^{-9}
2933	lipid hydroxylation	444.6	2.0x10^{-8}
70989	oxidative demethylation	323.4	7.6x10^{-8}
42178	xenobiotic catabolic process	142.3	2.4x10^{-6}
42573	retinoic acid metabolic process	131.7	2.8x10^{-6}
52695	cellular glucuronidation	149.8	1.0x10^{-4}
52697	xenobiotic glucuronidation	426.8	7.7x10^{-4}
