# Supplementary material for: Gut microbiota mediates SREBP-1c-driven hepatic lipogenesis and steatosis in response to zero-fat high-sucrose diet
Source: Mol Metab. 2025 May 7;97:102162. doi: 10.1016/j.molmet.2025.102162 (PMC12145984; doi:10.1016/j.molmet.2025.102162)
Supplement: Multimedia component 4 [file mmc4.pdf]

Supplementary Table 4. Concentration of hepatic triglyceride-derived fatty acids (nmol/mg) in *Srebf*-ASO and control-ASO mice. Related to Figure 4.

Fatty acid	*Srebf*-ASO	Control-ASO
FA 14:0	0.092 ± 0.043	0.12 ± 0.043
FA 16:0	3.87 ± 1.40	6.5 ± 2.3
FA 16:1	0.84 ± 0.35	1.1 ± 0.32
FA 18:0	0.61 ± 0.24	1.1 ± 0.49
FA 18:1	7.06 ± 2.93	13 ± 4.7
FA 18:2	0.17 ± 0.051	0.23 ± 0.054
FA 20:3	0.046 ± 0.011	0.056 ± 0.019
FA 20:5	0.00054 ± 0.00036	$0.0040 \pm 0.0014^{0.002}$
FA 22:6	0.0093 ± 0.0097	0.014 ± 0.012

n = 5 (*Srebf*-ASO); 4 (Control-ASO); Significant p-value determined by two-sided Students T test is displayed as superscript in the Table. Data are presented as mean ± SD.
