# Supplementary material for: Gut microbiota mediates SREBP-1c-driven hepatic lipogenesis and steatosis in response to zero-fat high-sucrose diet
Source: Mol Metab. 2025 May 7;97:102162. doi: 10.1016/j.molmet.2025.102162 (PMC12145984; doi:10.1016/j.molmet.2025.102162)
Supplement: Multimedia component 5 [file mmc5.pdf]

Supplementary Table 5. qRT-PCR primers used in the study.

Gene symbol	Direction	Sequence
Srebf1	Forward	AGCCATGGATTGCACATTTGA
Srebf1	Reverse	CAAATAGGCCAGGGAAGTCA
Fasn	Forward	TGGTGAATTGTCTCCGAAAAGA
Fasn	Reverse	CACGTTCATCACGAGGTCATG
Elovl6	Forward	ACAATGGACCTGTCAGCAAA
Elovl6	Reverse	GTACCAGTGCAGGAAGATCAGT
Scd1	Forward	CCGGAGACCCTTAGATCGA
Scd1	Reverse	TAGCCTGTAAAAGATTTCTGCAAACC
Mlxipl	Forward	CGGGACATGTTTGATGACTATGTC
Mlxipl	Reverse	CATCCCATTGAAGGATTCAAATAAA
Rpl32l	Forward	CCTCTGGTGAAGCCCAAGATC
Rpl32l	Reverse	TCTGGGTTTCCGCCAGTTT
