# Supplementary material for: Gut microbiota mediates SREBP-1c-driven hepatic lipogenesis and steatosis in response to zero-fat high-sucrose diet
Source: Mol Metab. 2025 May 7;97:102162. doi: 10.1016/j.molmet.2025.102162 (PMC12145984; doi:10.1016/j.molmet.2025.102162)
Supplement: Multimedia component 6 [file mmc6.pdf]

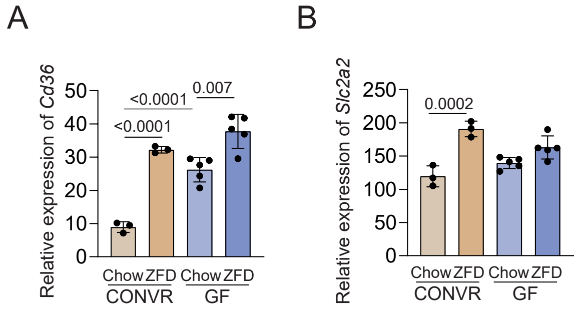

Supplementary Figure 1. Hepatic expression of *Cd36* and *Slc2a2*. RNA-seq analysis was performed on liver tissue from CONVR (n=3/diet) and GF (n=5/diet) mice fed ZFD or chow diet for 3 weeks. (A) Expression of *Cd36*. (B) Expression of *Slc2a2*. Adjusted p values for diet and colonization status were determined by two-way ANOVA with Tukey's multiple comparisons. Data are presented as mean ± SD. Abbreviations: CONVR – conventionally raised; GF – germ-free; ZFD – zero-fat diet; *Cd36* – cluster of differentiation 36; *Slc2a2* – solute carrier family 2 member 2

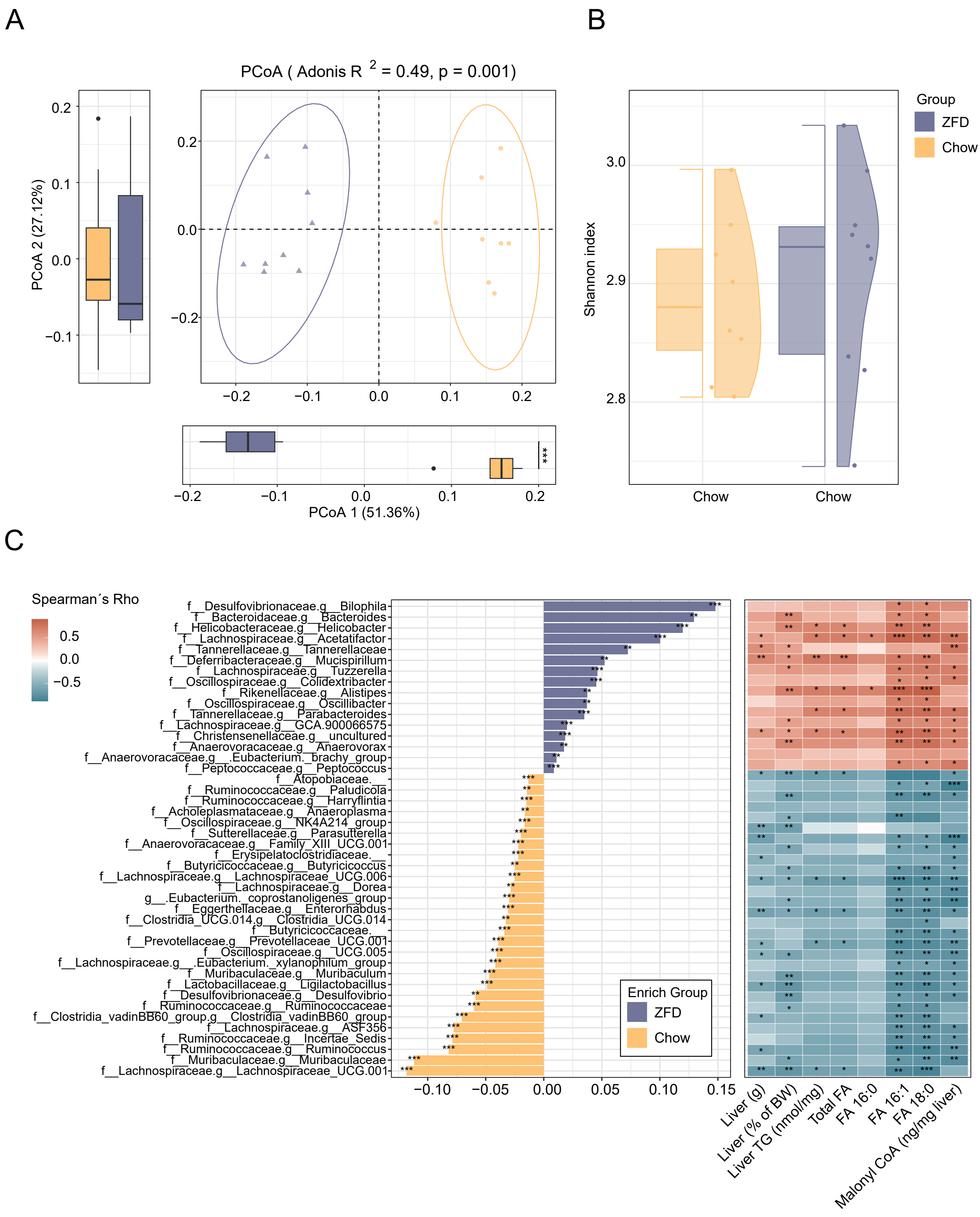

Supplementary Figure 2. 16S rRNA gene analysis of cecum microbiota in mice fed ZFD or chow diet. (A) Principal Coordinate Analysis (PCoA) on Bray-Curtis distance for mice fed chow (light yellow, n=8) or ZFD (light purple, n=9). The PCoA plot shows significant separation between the groups (Adonis R^2=0.49, p=0.001). Boxplots of the first two principal coordinates (PCoA1 and PCoA2) are shown alongside. (B) α-diversity comparison between groups using the Shannon Index (Wilcoxon Rank Sum test, p=0.54). (C) Differentially abundant taxa between groups. Taxa enriched in chow and ZFD are colored in light yellow and light purple, respectively (MaAsLin2, adjusted $p<0.01$). The panel on the right shows Spearman correlations between the differentially abundant genera and various liver and metabolic parameters. *** $p < 0.001$, ** $p < 0.01$, * $p < 0.05$.

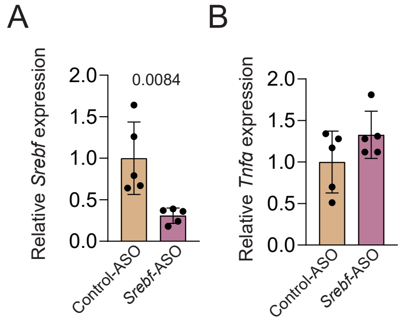

Supplementary Figure 3. *Srebf1* and *Tnfα* expression in the liver of CONVR mice treated with *Srebf1*-specific ASO. Mice were fed ZFD or chow diet for 3 weeks. Gene expression was determined by qRT-PCR (n=5/group). (A) *Srebf1* expression and (B) *Tnfα* expression. *P* values were determined by Student's *t*-test. Data are presented as mean ± SD. Abbreviations: CONVR – conventionally raised; ZFD – zero-fat diet; *Srebf1* – sterol regulatory element binding transcription factor 1; *Tnfα* – tumor necrosis factor alpha; ASO – antisense oligonucleotide.
